# CD24 Positive Nucleus Pulposus Cells in Adult Human Intervertebral Discs Maintain a More Notochordal Phenotype Than GD2 Positive Cells

**DOI:** 10.1002/jsp2.70029

**Published:** 2024-12-23

**Authors:** Andra‐Maria Ionescu, Pauline Baird, Sonal Patel, Gareth Howell, Judith A. Hoyland, Stephen M. Richardson

**Affiliations:** ^1^ Division of Cell Matrix Biology and Regenerative Medicine, School of Biological Sciences, Faculty of Biology, Medicine and Health University of Manchester, Manchester Academic Health Sciences Centre Manchester UK; ^2^ Flow Cytometry Core Facility, Faculty of Biology, Medicine & Health University of Manchester Manchester UK; ^3^ Manchester Cell‐Matrix Centre, School of Biological Sciences, Faculty of Biology, Medicine and Health University of Manchester, Manchester Academic Health Sciences Centre Manchester UK

**Keywords:** CD24, cell sub‐populations, flow cytometry, GD2, intervertebral disc, notochordal, nucleus pulposus

## Abstract

**Background:**

Notochordal cells (NCs) present in the nucleus pulposus (NP) of the developing human intervertebral disc (IVD) disappear during the first decade of life. This loss coincides with the onset of IVD degeneration, therefore these cells are hypothesized to be important in NP homeostasis. Putative NC‐derived (CD24^+^) and progenitor (TIE2^+^/GD2^+^) cell sub‐populations have previously been identified in the adult human NP, but their characteristics have yet to be compared. Here, we used CD24, TIE2 and GD2 to identify and then isolate discrete cell sub‐populations to assess cell phenotype.

**Methods:**

CD24, GD2 and TIE2 positivity was assessed in a cohort of human pediatric and adult NP samples across a range of ages and histological degeneration grades using immunohistochemistry and flow cytometry. FACS sorting was used to isolate different cell sub‐populations (CD24^+^/GD2^+^; CD24^+^/GD2^−^; CD24^−^/GD2^+^; CD24^−^/GD2^−^). Cell phenotype was assessed using qPCR for known NC and NP markers as well as catabolic genes.

**Results:**

CD24^+^ and GD2^+^ cells were localized in all samples, irrespective of age or degeneration grade, while TIE2^+^ cell number was consistently very low. The same positivity trend was confirmed using flow cytometry. A small CD24^+^/GD2^+^ sub‐population was present and maintained marker expression with time in culture. CD24^+^ subpopulations showed a significantly higher expression of NC markers than the CD24^−^ subpopulations and unsorted samples, suggesting a healthier phenotype in the CD24^+^ cells. GD2 did not appear to influence gene expression.

**Conclusions:**

This study provides a better understanding of different cell sub‐populations present in the adult NP, with identification of CD24^+^/GD2^+^ cells that are maintained with aging and degeneration. Healthy, NC‐like phenotypic profiles appeared reliant on CD24, rather than GD2. The study highlights the importance of studying discrete cell sub‐populations, especially CD24^+^ NP cells to better understand their role in NP homeostasis.

## Graphical abstract

This study characterizes cell sub‐populations within the adult human intervertebral disc based on the expression of CD24, GD2 and TIE2. The findings highlight the importance of studying discrete cell sub‐populations, especially CD24+ NP cells to better understand their role in NP homeostasis.

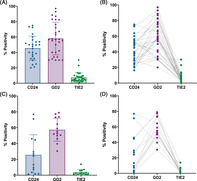

## 1 Introduction

Chronic low back pain (LBP) is one of the leading causes of years lived with disability and has a high socioeconomic impact globally [1]. The etiology of LBP is complex and multifactorial, with factors such as age, occupation, genetics, weight and posture all contributing to the symptoms of a particular patient [2]. It has been shown that intervertebral disc (IVD) degeneration is a major contributor to more than 50% of cases of LBP [2]. During disc degeneration cells within the central nucleus pulposus (NP) region of the IVD increase their expression of pro‐inflammatory cytokines such as IL‐1β and TNFα, which in turn drives increases in expression of matrix degrading enzymes, including members of the matrix metalloproteinase (MMP) and a disintegrin and metalloproteinase with thrombospondin motifs (ADAMTS) families, and a decrease in expression of key matrix molecules such as aggrecan and collagen type II [3, 4].

The cells present within the fetal human NP are large and vacuolated cells and lineage tracing studies in mice have demonstrated that they are derived from the embryonic notochord [5]. With skeletal maturity in humans there is a loss of these identifiably large, vacuolated notochordally‐derived cells (NC cells). Instead, the tissue contains a population of smaller, round and non‐vacuolated chondrocyte‐like cells which correspond to the adult NP cells [6].

Studies in canine models showed that in chondrodystrophic dogs, replacement of NC cells with NP cells coincides with onset of disc degeneration and these dogs had a higher prevalence and earlier onset of IVD degeneration. Conversely, in non‐chondrodystrophic dogs, NC cells persist throughout life and are believed to confer resistance to and delay the onset of disc degeneration, especially in earlier years [7, 8, 9]. Other studies have shown that notochordal cells secrete soluble factors that can induce anti‐inflammatory, pro‐anabolic and anti‐cell death effects upon NP cells [10, 11, 12, 13, 14], thus supporting the importance of NC cells in IVD health maintenance and delay of degeneration. Harnessing the regenerative potential of these cells could lead to new cellular and molecular strategies in the treatment of human IVD degeneration. However, to achieve this, it is important to fully understand the phenotype of cells within the adult human NP to allow a better understanding of the physiology and function of these cells.

There are an increasing number of studies shedding light on the heterogeneity of the human NP by revealing the existence of distinct cell sub‐populations within the human adult NP [15, 16, 17, 18, 19, 20, 21, 22, 23, 24, 25, 26]. We previously identified a small sub‐population of cells within the adult human NP with an NC‐like phenotype expressing the NC markers carbonic anhydrase 12 (CA12), galectin 3 (GAL3), brachyury (TBXT) and CD24 [18]. These CD24^+^ NP cells have also been shown to possess a more favorable phenotype compared to CD24^−^ cells which were more prone to senescence, expressed higher levels of MMPs and lower levels of ACAN and COL2, suggesting loss of CD24 may be linked to matrix breakdown and reduced formation of the main ECM components [27]. Likewise, further investigation of human and rat NP suggested that CD24^+^ cells may represent a notochordally‐derived cell subpopulation as they expressed multiple notochordal cell‐associated markers (brachyury, SHH and GLUT‐1) [28].

Sakai et al., have also proposed hierarchical subsets of NP cells, with TIE2^+^/GD2^+^ cells representing a ‘progenitor‐like’ population, with loss of TIE2 and adoption of CD24 delineating commitment to an NP phenotype [16]. TIE2^+^ cells have subsequently been isolated from the adult human disc with an age‐dependent decrease is expression identified [29], while TIE2^+^/GD2^+^ cell populations have subsequently been shown to express known NC markers including T, aggrecan and type II collagen [30].

While all three markers (CD24, TIE2 and GD2) have been proven to be expressed by cell sub‐populations in the adult human NP, it is yet to be established whether cells in the adult human NP expressing CD24 also co‐express TIE2 and GD2 or whether these are different subpopulations of cells. Thus, the aims of this study were firstly to identify cells expressing CD24, TIE2 and GD2 within cohorts of pediatric and adult human IVD using immunohistochemistry and flow cytometry; then secondly, to determine whether using a combination of these markers could improve on the ability to isolate discrete cell subpopulations from the adult NP; and lastly, to assess the phenotype of the sub‐populations identified by studying their expression of known notochordal/healthy NP marker and catabolic genes.

## 2 Methodology

### 2.1 Sample Acquisition and Processing

All experiments and procedures carried out in this study were performed with relevant approval from the UK National Research Ethics Service and The University of Manchester and with written, informed consent from adult patients undergoing surgery for chronic back pain, or pediatric patients undergoing surgery for idiopathic scoliosis. NP tissue from each sample was macroscopically dissected from the surrounding structures, if present in the sample, and further processed for cell extraction and/or fixation. A summary of donors and their use in the study is detailed in Table 1.

**TABLE 1:** Human disc samples used for experiments as tissue and/or cells. Degeneration score based on histological grading. • represents samples used for IHC and/or flow cytometry analysis. ▪ represents samples used for FACS.

Degeneration Grade	Patient age	Sex	IHC (tissue)	Flow/FACS (cells)
Pediatric				
0	10y	F	•	
0	13y	F	•	
0	14y	F	•	
0	15y	F	•	
0	16 y	F	•	
0	17 y	M	•	
Adult				
3	52y	F	•	
3	43y	F	•	
3	71y	F	•	
4	26y	F	•	
4	28y	F	•	
4	53y	F	•	
4	35y	F	•	
4	46y	F	•	
4	75y	F	•	
5	53y	M	•	
5	28y	F		•
5	33y	M		• / ▪
5	40y	F	•	•
5	74y	M		• / ▪
6	35y	F	•	
6	56y	F	•	
6	57y	F		•
7	46y	F	•	
7	34y	F		• / ▪
7	24y	F	•	•
7	27y	M		•
7	28y	F		•
7	39y	F		•
7	27y	F	•	
7	42y	F	•	
7	73y	F	•	
7	79y	F	•	
8	18y	M	•	
8	22y	M	•	
9	35y	M		•
9	53y	M		•
10	20y	F	•	
10	32y	F	•	
10	37y	F		•
11	53y	M	•	
11	47y	F	•	
11	72y	F	•	
11	74y	F	•	
12	60y	M		•
12	65y	M	•	
12	74y	F	•	
12	76y	F	•	

For immunohistochemistry, a portion of NP tissue was fixed in 4% (w/v) paraformaldehyde/PBS and processed to paraffin wax. Five‐micron sections were cut, stained with H&E and histologically graded by an experienced histopathologist using a previously described scale from 0 (non‐degenerate) to 12 (severely degenerate) [31].

For NP cell extraction, enzymatic digestion was performed by incubating the minced tissue in 10 mL of 0.1% (w/v) collagenase type II (260 units/mg, Gibco, 17 101–015) in DMEM‐high glucose (Sigma‐Aldrich, D0819) media supplemented with 100 U/mL penicillin, 100 μg/mL streptomycin, 0.25 μg/mL amphotericin (Sigma), 10 μM ascorbic acid‐2 phosphate (Sigma), and 100 mM sodium pyruvate (Sigma), for 2–3 h at 37° with agitation. The obtained solution was filtered through a 40 μm strainer and centrifuged at 400 g for 5 min. If the pellet contained blood cells, this was resuspended in 3 mL of red blood cell lysis buffer (155 mM NH_4_Cl (8.29 g/L), 10 mM KHCO_3_ (1.01 g/L) and 1 mM NaEDTA (0.744 g/L) diluted in deionized water up to 1 L) and incubated at 37° for 10 min with agitation and then diluted with 7 mL of described media and recentrifuged. Cells were then cultured for 1–6 passages on tissue culture treated polystyrene, in media described above with added 10% v/v fetal bovine serum (Sigma). Cells were grown in ambient incubator conditions (37°C, 5% CO_2_, ambient O_2_).

### 2.2 Immunohistochemistry

Expression of CD24, TIE2 and GD2 was assessed using immunohistochemistry on 5 μm paraffin sections of pediatric and adult NP tissue. Heat‐induced epitope retrieval (HIER) was used (steamer) and performed prior to the endogenous peroxidase blocking step. HIER buffer included 80 mL of 10 mM citric acid at pH 6.0 which was made up to 800 mL with deionized water and heated in a microwave. Slides were then immersed in the buffer and placed in the steamer for 10 min, followed by another 10 min incubation on the benchtop. Slides were then blocked for endogenous peroxidase by immersing slides into 100% IMS containing 0.3% (v/v) hydrogen peroxide and 25 mM HCl for 30 min at room temperature (RT). Sections were then rinsed in deionized water and washed in tris buffered saline (TBS) 2 × 5 min. Non‐specific binding sites were blocked with 25% goat serum (Sigma, G9023) in TBS containing 1% w/v bovine serum albumin (BSA, Sigma, A9647) at RT for 30 min.

Sections were incubated overnight at 4° with the following pre‐optimized primary antibodies: rabbit CD24, (1:100) (Abcam, ab199140), mouse Tie2 (1:15) (Novus Biologicals, NB110‐60986) and rabbit GD2 (1:2000) (Matreya LLC, #1963) in 1% BSA in TBS. Positive control tissues, secondary antibody only and isotype control (Rabbit IgG (I‐1000‐5) and Mouse IgG (I‐2000‐1), Vector Laboratories) sections were also assessed. Slides were washed 3 × 5 min in TBS from this point until the end of the protocol between each step, unless otherwise stated. Secondary antibody incubation was performed using goat anti‐mouse for Tie2 (Goat Anti‐Mouse IgG Antibody (H + L), biotinylated (BA‐9200‐1.5), Vector Laboratories) and goat anti‐rabbit for CD24 and GD2, 1:300 (Goat Anti‐Rabbit IgG (H + L), Biotinylated, BA‐1000‐1.5, Vector Laboratories) for 1 h at RT. Amplification was performed by incubating with 4x drops/slide of ABC‐Amplification reagent (Vectastain) for 30 min at room temperature. The avidin‐biotin complex was detected with the 3,3'‐Diaminobenzidine (DAB) Liquid Substrate System (Sigma, D7304), where 1 drop DAB Liquid Chromogen was mixed with 1 mL of ready‐to‐use peroxide solution. Sections were incubated in the buffer at RT for 10 min. Sections were then washed in deionized water for 4 min and nuclei were counterstained by incubating for 2.5 min in freshly filtered Mayer's hematoxylin. Excess staining was removed by rinsing slides in running tap water for 5 min. Stained sections were finally dehydrated in IMS for 4 × 5 min, cleared in xylene for 3 × 5 min and mounted with a coverslip in mounting medium (DPX).

Immunohistochemistry‐stained sections were scanned using the Pannoramic 250 Flash II digital slide scanner (3DHistech) and visualized using the Caseviewer software (3DHistech). Positive cell quantification was performed for each marker using the Qupath software. Percentage positivity was calculated as number of positive cells out of total number of NP cells in the tissue and graphs were produced using GraphPad Prism v10. An Ordinary one‐way ANOVA, with a test for linear trend was performed to assess significance and *p* < 0.05 was considered significant.

### 2.3 FACS/ Flow Cytometry

A minimum of 10 000 cells per sample were incubated in primary antibodies (Table 2) or isotype control diluted in FACS Buffer (Ca^2+^/Mg^2+^‐free PBS, 5 mM EDTA and 1% BSA). Samples were incubated in the dark for 15 min and then washed in FACS Buffer and centrifuged for 10 min at 300 g at 4°C. Cells were resuspended in 300 mL of FACS Buffer and either analyzed using a BD Fortessa X20 flow cytometer and FlowJo software or sorted using a BD Influx (BD Biosciences) cell sorter. Sorted cells were collected in media described above containing 20% (v/v) FBS and returned to culture.

**TABLE 2:** Antibody details, supplier details and dilutions used for flow cytometry and FACS.

Antibody details	Supplier details	Dilution
Mouse Anti‐Human CD24‐PE	BD Pharmingen Cat no: 555428	1:20
Mouse Anti‐Human Disialoganglioside (GD2)‐BV421	BD Pharmingen Cat no: 564223	1:20
Mouse Anti‐Human Tie2 (CD202b)‐Alexa Fluor 647	BD Pharmingen Cat no: 566716	1:20
Mouse IgG2a, κ‐PE (Isotype Control)	BD Pharmingen Cat no: 555574	1:40
Mouse IgG2a, κ‐BV421 (Isotype Control)	BD Horizon Cat no: 562439	1:100
Mouse IgG1κ‐Alexa Fluor 647 (Isotype Control)	BD Pharmingen Cat no: 565571	1:40

### 2.4 Cell Kinetics Analysis

Cells were counted before each passage and 3 × 10^5^ were seeded at the start of each subsequent passage. Cell number and number of days between passages were recorded and used to calculate cumulative population doublings using the formula:

$$\mathrm{PD}={\mathrm{PD}}_{0}+3.322\;\left(\log C_{f}-\log C_{i}\right)$$

where PD = population doublings, PD_0_ = population doublings at time 0, 3.322 = proliferation rate constant, *C*_f_ = final cell number, and *C*_i_ = initial cell number.

### 2.5 Real‐Time PCR Analysis

RNA was extracted at P0 or P1 using TRIzol according to the manufacturers protocol and resuspended in 20 μL TE buffer. Concentration and purity were determined using Nanodrop spectrophotometer and then the ABI high‐capacity cDNA reverse transcription kit and manufacturers protocol (ThermoFisher) was used to convert sample RNA to cDNA. Real‐time quantitative PCR (qPCR) was used to determine the gene expression of target genes (Table 3). Samples were loaded in triplicate into 96‐well plates, with each well containing 8 μL reaction mixture containing: 5 μL SYBR Fast Mastermix (FastStart SYBR Green Master, Sigma) 1 μL each forward (F) and reverse (R) primers and 1 μL molecular grade water, plus 2 μL of sample cDNA (5 ng/μl). Positive (human genomic DNA at 5 ng/μl) and negative (molecular grade water) controls were also included. Plates were run on an ABI StepOne Plus real‐time PCR machine. Data was analyzed using 2^−ΔCt^ by calculating the relative expression of the target gene to the reference gene GAPDH. qPCR was performed on all samples used for cell sorting. Statistical analysis was performed using Graphpad Prism 10 software and ordinary one‐way ANOVA with multiple comparisons test was applied. Multiple comparisons were made between each experimental group and determined as significant if *p* < 0.05.

**TABLE 3:** Details of target genes and SYBR Green primer sequences used for qPCR.

Gene	Primer sequence	Final primer concentration (nM)	Accession number	Amplicon size
GAPDH	F: CTCCTCTGACTTCAACAG R: CGTTGTCATACCAGGAAA	600	NM_001256799	104
SOX9	F: GCTCTGGAGACTTCTGAA R: GGTACTTGTAATCCGGGTG	450	NM_000346	101
COL2A1	F: GGCTTCCATTTCAGCTATG R: CAGTGGTAGGTGATGTTC	450	NM_033150	167
ACAN	F: GGCTTCCACCAGTGTGAC R: GTGTCTCGGATGCCATACG	900	NM_001135	131
TBXT	F: CTCACCAACAAGCTCAAC R: CTGTGATCTCCTCGTTCTG	900	NM_003181	172
CD24	F: GCTCCTACCCACGCAGATTTAT R: CCTTGGTGGTGGCATTAGTTG	900	NM_001359084	116
KRT8	F: CCTCATCAAGAAGGATGTG R: CCTGAGGAAGTTGATCTC	600	NM_002273	94
KRT18	F: CTGCTGAACATCAAGGTC R: AGGCATCACCAAGATTAAAG	600	NM_199187	91
KRT19	F: CCATGAGGAGGAAATCAGTA R: GTCACTCAGGATCTTGGC	450	NM_002276	103

## 3 Results

### 3.1 Immunohistochemical Expression of CD24, GD2 and TIE2 With Aging and Degeneration

**FIGURE 1:**
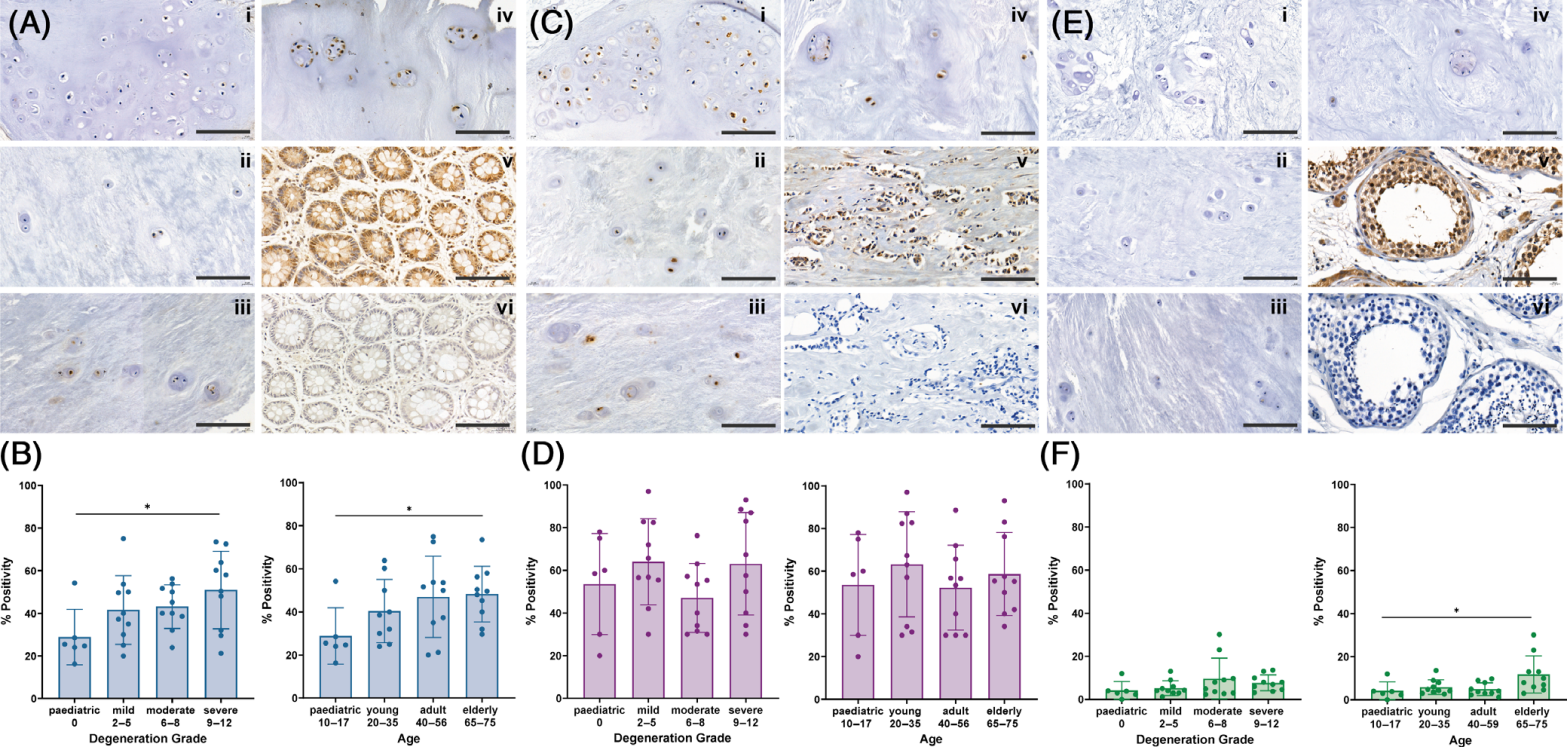
CD24, GD2 and TIE2 immunostaining in a cohort of pediatric and adult NP samples of a range of ages and degeneration grades. Representative images showing immunostaining for CD24 (A), GD2 (C) and TIE2 (E). Scale bars = 100 μm. For each marker, images represent (i) non‐degenerate (grade 0), pediatric NP tissue (10 years old), (ii) mild degeneration (grade 4), adult NP tissue (28 years old); (iii) moderate degeneration (grade 7), elderly NP tissue (73 years old); (iv) severe degeneration (grade 12), elderly NP tissue (65 years old), (v) positive control (CD24: Colon; GD2: Breast carcinoma; TIE2: Testis) and (vi) IgG control (CD24: Colon; GD2: Breast carcinoma; TIE2: Testis). Quantification of marker positivity: (B) CD24, (D) GD2 and (F) TIE2, based on degeneration grade (B, D, F): Pediatric (degeneration grade = 0; *n* = 6), mild degeneration (2–5; *n* = 10), moderate degeneration (6–8; *n* = 10), severe degenerate (9–12; *n* = 10) and age (A, C, E): Pediatric (10–17, *n* = 6), young (20–35; *n* = 10), adult (40–56; *n* = 10); and elderly (65–75; *n* = 10). Graphs show mean ± SD. *p < 0.05, one‐way ANOVA, multiple comparisons, test for linear trend, post hoc test.

Immunostaining of pediatric (*n* = 6) and adult IVD tissue (*n* = 30) revealed sub‐populations of cells positive for CD24 and GD2, as well as small subpopulations of cells positive for TIE2, throughout aging and degeneration (Figure 1). No difference in staining pattern was observed between different degeneration grades and ages (Figure 1A,C,E), with staining visible in the cell membrane and cytoplasm. CD24 staining appeared in both single cells and clusters, with staining intensity appearing highest in clustered cells in severely degenerate tissue. Likewise, GD2 expression was observed in both single cells and clusters, with intense staining observed in pediatric clustered cells. TIE2 expression was only observed in small numbers of cells and no clear trend could be observed between single cells and cells within clusters.

CD24 and GD2 expression was found in all samples analyzed, with the number of positive staining cells varying with age and degeneration, particularly for the CD24 marker (Figure 1B,D). Pediatric samples had the lowest number of CD24 positive cells (mean 28.9%; range 16.3%–54.2%) and the high degeneration grade samples the highest number (mean 50.2%; range 21.2%–73.5%).

Although there was no significant difference in CD24 expression between different grades of degeneration, there was a significant linear trend (*p* = 0.022) between the group means, in pediatric to high degeneration grade order (Figure 1B). Similarly, when comparing different age groups, the pediatric samples had on average the lowest CD24 cell number positivity (mean 28.9%; range 16.3%–54.2%) and the elderly adults the highest (mean 47.18%; range 29.6%–73.5%), with a significant linear trend (*p* = 0.034) between the group means, in pediatric to elderly adult order (Figure 1B).

A varied, but generally high GD2 positivity was observed in all samples, with no significant difference between the different degeneration grade and age groups (Figure 1D). While TIE2 cell positivity was observed in all samples, expression was consistently low, with no significant difference in staining between the different degeneration grade and age groups (Figure 1F). However, a significant linear trend (*p* = 0.011) was identified between group means, increasing from pediatric (mean 4.1%; range 0.0%–12.0%) to elderly adult (mean 11.8%; range 3.0%–30.1%) (Figure 1F). All positive controls showed staining in expected cell populations and both no primary and IgG controls were negative.

### 3.2 CD24, GD2, TIE2 Expression Differences at Cell and Tissue Level

**FIGURE 2:**
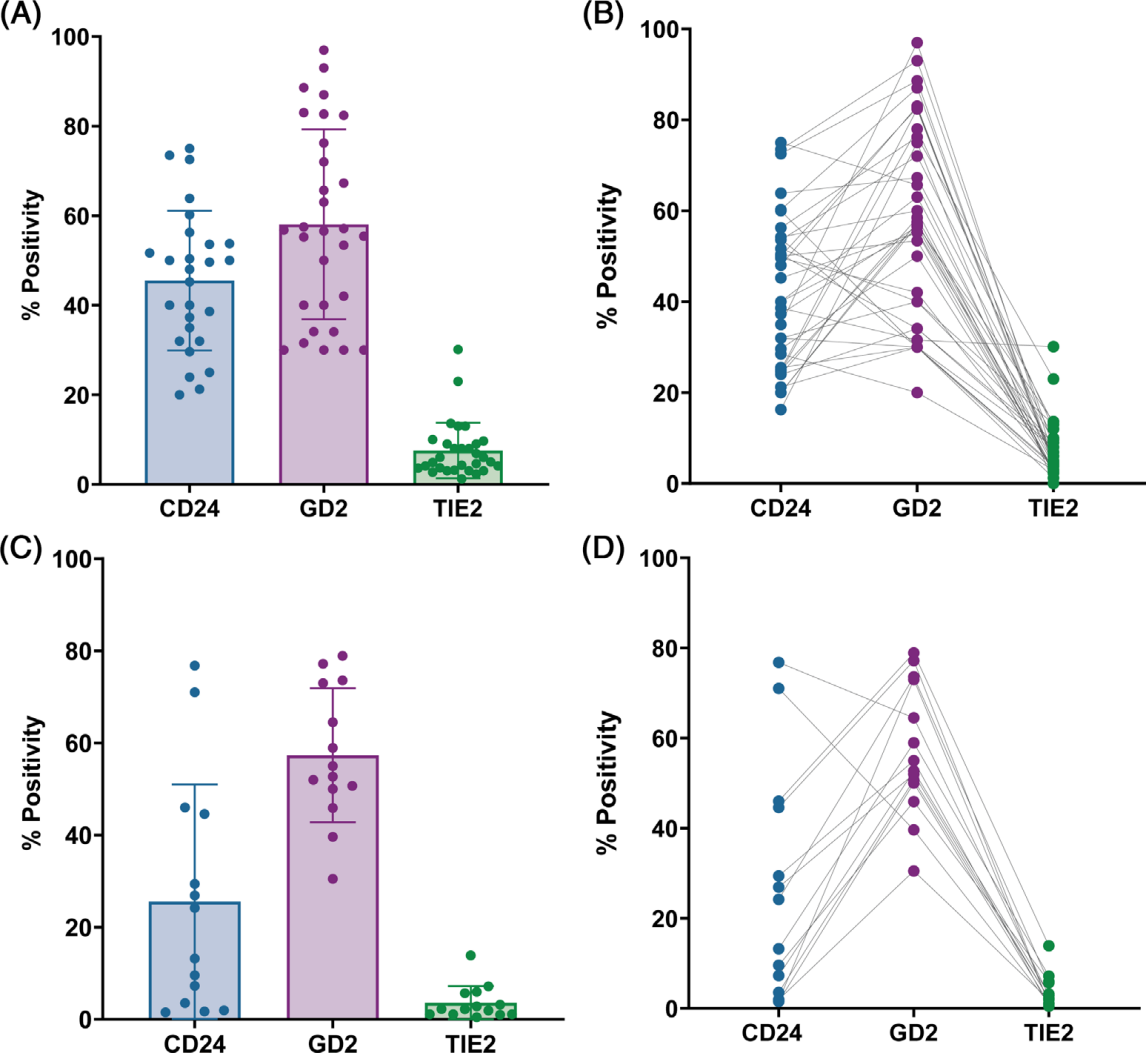
Quantification of CD24, GD2 and TIE2 positive cells in human primary adult NP samples. (A) Bar graph showing mean (±SD) CD24, GD2 and TIE2 positivity in tissue determined using immunostaining (*n* = 30) and (B) line graphs comparing immunopositivity in each sample. (C) Bar graph showing mean (±SD) CD24, GD2 and TIE2 positivity in NP cells at passage 1 of culture determined using flow cytometry (*n* = 14) and (D) line graphs comparing cell positivity in each sample.

Overall immunopositivity of CD24, GD2 and TIE2 revealed 45.5% (range 20%–75%) of cells were positive for CD24 irrespective of patient age or grade of degeneration, whilst 58.1% (range 30%–97%) of cells were positive for GD2 and only 7.5% (range 1.2%–30.1%) were positive for TIE2 (Figure 2A). In most cases, GD2 positivity was higher than CD24, whilst all samples always demonstrated lower levels of TIE2 immunopositivity than either CD24 or GD2 (Figure 2B).

Expression of CD24, GD2 and TIE2 was also assessed using flow cytometry in P1 primary adult human NP cells (Figure 2C,D). CD24 positivity (mean 25.6%; range 1.5%–76.8%) was lower than seen in tissue, but the percentage of cells positive for GD2 (mean 57.3%; range 30.5%–78.9%) and TIE2 positivity (mean 3.5%; range 0.5%–13.9%) with flow cytometry was similar to that seen in immunostaining. Likewise, the trend for marker expression was similar between immunolocalization and flow cytometry, with most samples showing higher GD2 and lower TIE2 compared to CD24.

### 3.3 Isolating Sub‐Populations of Cells Based on CD24 and GD2 Expression

Since very low TIE2 positivity was observed in both tissue‐resident cells and isolated cells in culture, samples were sorted based only on CD24 and GD2 expression. Human primary adult NP samples (P1; *n* = 3) were sorted into four sub‐populations: CD24^−^/GD2^−^, CD24^−^/GD2^+^, CD24^+^/GD2^−^, CD24^+^/GD2^+^.

**FIGURE 3:**
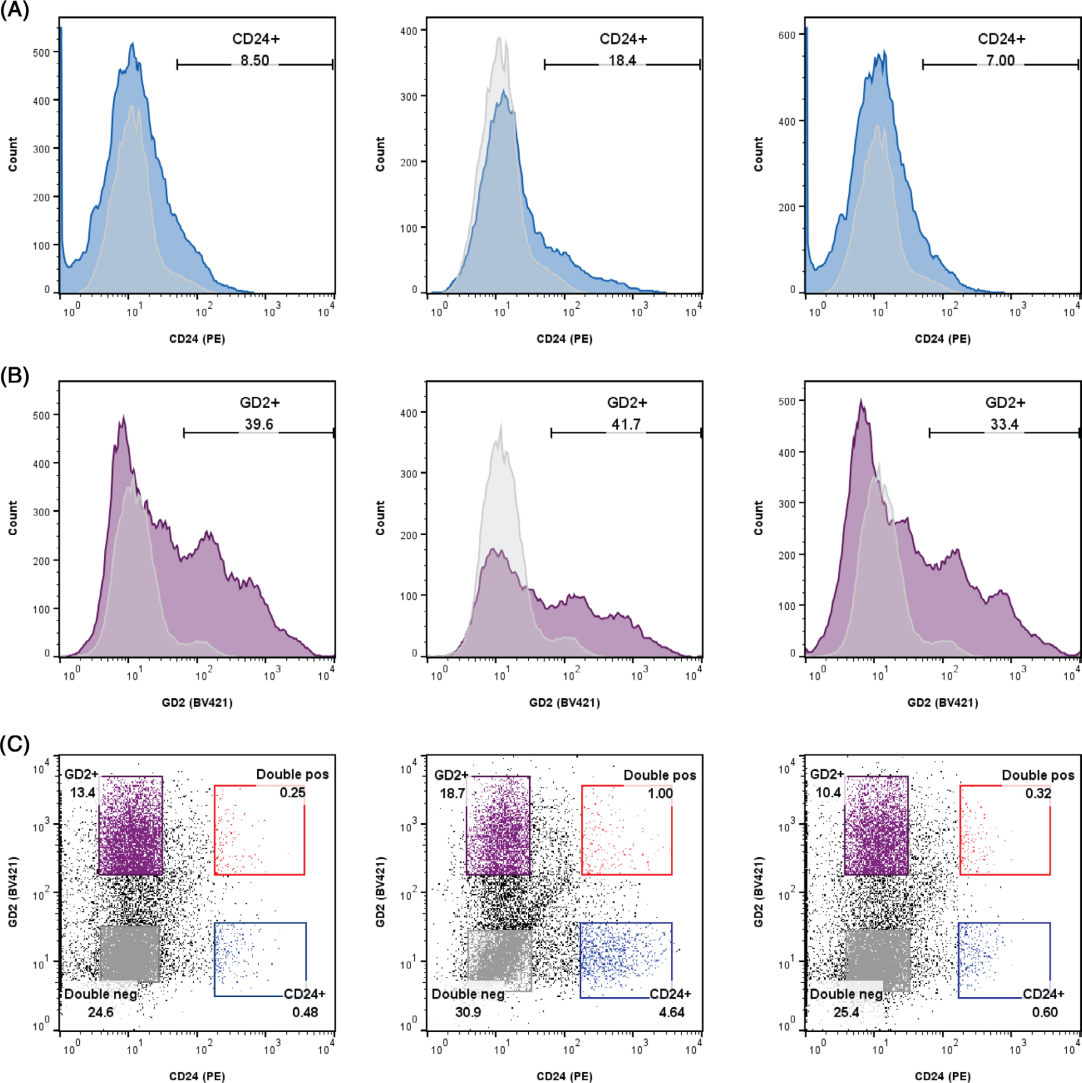
Flow cytometry data of sorted sub‐populations from human adult NP samples (*n* = 3). Histograms showing (A) CD24 and (B) GD2 positivity in each of the three samples. Gray plots represent isotype controls. (C) FACS gating for the four sub‐populations at P1.

A relatively low CD24 positivity of 7%–18.4% (Figure 3A) and higher GD2 positivity of 33.4%–41.7% (Figure 3B) was found in the three samples selected for sorting. To ensure the homogeneity of the sorted populations, stringent gating was used (Figure 3C). Due to the relatively low number of CD24^+^ cells at the time of sort, following gating, very few cells were sorted for the CD24^+^ populations, 0.51%–4.64% for the CD24^+^/GD2^−^ population and 0.25%–1% for the CD24^+^/GD2^+^ population (Figure 3C).

### 3.4 CD24 ^+^ Sorted Cells Display a More Anabolic and Notochordal Phenotype Than CD24 ^−^ Sorted Cells

**FIGURE 4:**
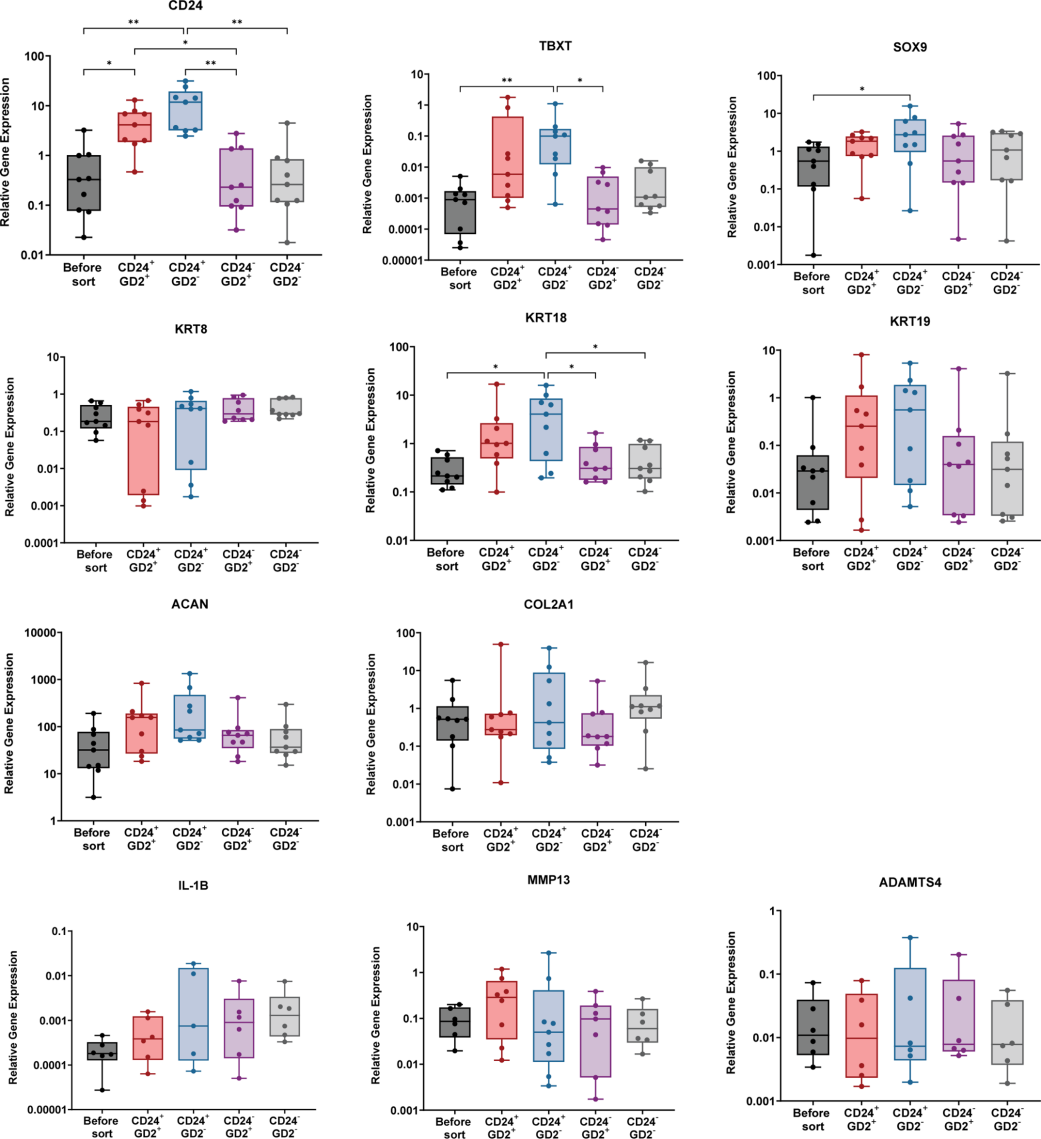
qPCR results showing gene expression changes of known notochordal, extracellular matrix and catabolic markers in human primary adult NP cells before sorting and in the four sorted populations (*n* = 9). Data was normalized to GAPDH and presented as 2^−ΔCt^ values. Data shown as box & whisker graphs, min to max; *p < 0.05, one‐way ANOVA, multiple comparisons.

The qPCR analysis demonstrated that CD24^+^ sub‐populations (CD24^+^/GD2^−^ and CD24^+^/GD2^+^) generally showed a higher gene expression and broader range of expression of known notochordal and healthy NP markers when compared to the mixed pool (unsorted cells) or to the CD24^−^ sorted sub‐populations (CD24^−^/GD2^−^ and CD24^−^/GD2^+^) (Figure 4). The CD24^+^/GD2^−^ population had a significantly higher expression of notochord development genes TBXT and SOX9 and healthy NP gene KRT18 compared to the unsorted cells and CD24^−^ populations. Moreover, CD24^+^ populations generally showed a broader range of expression compared to the CD24^−^ and unsorted populations for genes such as TBXT (CD24^+^/GD2^+^), COL2A1 (CD24^+^/GD2^−^), KRT8 and KRT19. Conversely, GD2 positivity alone did not appear to affect the phenotype of NP cells, with the CD24^−^/GD2^+^ population showing similar notochordal and healthy NP gene expression to unsorted and CD24^−^/GD2^−^ cells. Expression of either CD24 or GD2 did not significantly affect expression of the catabolic genes IL‐1β, MMP13 and ADAMTS4.

### 3.5 CD24 And GD2 Expression in Sorted Sub‐Populations Is Not Stable With Time in Culture

**FIGURE 5:**
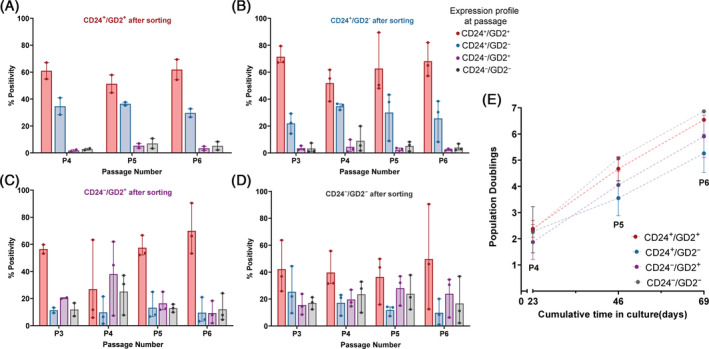
Quantification of cell positivity for CD24 and GD2 with time in culture post‐sorting (*n* = 3). Percentage positive for CD24 and GD2 alone or in combination at various passages following sorting of (A) CD24^+^/GD2^+^; (B) CD24^+^; (C) GD2^+^; (D) CD24^−^/GD2^−^ sub‐populations; (E) growth rates of the four sub‐populations.

CD24 and GD2 positivity was monitored with time in culture (P3‐P6) (Figure 5A–D). Due to the low cell numbers sorted for the CD24^+^/GD2^+^ population (Figure 3C), flow data was only available from P4 onwards (Figure 5A).

Although stringent gating was used, it was observed that the CD24^−^ populations (CD24^−^/GD2^−^ and CD24^−^/GD2^+^) became heterogeneous by P3 when marker expression was observed using flow cytometry, with CD24 and GD2 positivity found in the CD24^−^/GD2^−^ population (Figure 5A) and unexpected CD24 positivity found in the CD24^−^/GD2^+^ population (Figure 5B), positivity which was maintained until P6.

The CD24^+^ populations (CD24^+^/GD2^−^ and CD24^+^/GD2^+^) were more stable and maintained a high positivity of the markers with time in culture (Figure 5C,D). However, a level of heterogeneity was still observed. In the CD24^+^/GD2^−^ population, expected to only contain CD24^+^ cells, high GD2 positivity was observed as well, with over 50% of cells double positive for the markers across passages 3–6 (Figure 5C). Cells in the CD24^+^/GD2^+^ population, expected to express both markers, had similar expression profile to the CD24^+^/GD2^−^ population, with over 20% of cells losing GD2 positivity by P3 (Figure 5D).

When looking at the kinetics of the four populations, these presented with similar proliferation rates, suggesting marker expression did not influence cell proliferation (Figure 5E).

## 4 Discussion

We have previously shown that a sub‐population of human NP cells express the notochordal cell marker CD24 and that expression is maintained with aging and degeneration, supporting the hypothesis that there is a notochordal origin for at least a proportion of cells present within the mature human NP [18]. This persistence of cells with a notochordal‐like phenotype but with NP‐like morphology within the adult NP has been often reported in recent years, implying the possibility of cell sub‐populations within the adult NP [16, 17]. Conversely, TIE2^+^ and GD2^+^ cells have also been previously identified in mouse and human IVDs and have been used to isolate cell sub‐populations from adult degenerate NP samples [16, 30, 32]. Our aim was to study expression patterns for CD24, TIE2 and GD2 in pediatric and adult human NP cells and understand whether expression was linked with a specific cell phenotype.

Comparable to the results of previous studies, generally high GD2 and very low TIE2 positivity was seen in samples of different degeneration grades and ages in both cells within tissue and in cultured cells. The Sakai group [30] suggest that the age of the human donor is crucial when looking at TIE2 expression from extracted cells as it decreases drastically post adolescence (TIE2 positivity ranges from 70%–80% during the early 20s and suddenly decreases to under 20% by the mid‐20s). Interestingly, in this study, most samples analyzed displayed low TIE2 positivity (under 15%), regardless of age and degeneration grade and this included tissue from younger, non‐degenerate donors between 10 and 17 years of age. This suggests that TIE2 expression is highly unstable and is perhaps dependent on a range of variables including the technique used for assessment, age of the donor, whether or not the cells have been cultured and the duration and method of culture [30]. Since isolating sufficiently large populations of viable cell sub‐populations for further study is technically challenging, TIE2 was not included in the cell sorting studies.

CD24 and GD2 showed expression in both adult tissue‐resident and cultured cells of a range of degeneration grades and ages. The sequential “switching on” of GD2 and CD24 expression during culture suggests a potential hierarchy in the cell populations, a pattern of differentiation or maturation, where cells first switch on GD2 expression and then CD24 expression which is subsequently maintained. A similar cellular hierarchy was previously proposed by Sakai *et al*., 2012 [16] who hypothesized how the patterns of CD24 and GD2 expression change depending on the degree of cellular differentiation, where expression of GD2 increases during the differentiation of TIE2^+^ NP progenitor cells to NP‐committed cells and then decreases during the final stages of differentiation and expression of CD24 increases during the differentiation of the NP (NP‐committed cells). Moreover, gene expression results of known notochordal and healthy NP markers suggest that the presence of CD24, rather than GD2, leads to a healthier phenotype (such as increased TBXT, SOX9 and KRT18) in adult NP cells of different degeneration grades and ages. These results match those previously described where CD24^+^ NP cells were shown to have a healthier phenotype than CD24^−^ cells, with higher aggrecan and type II collagen expression [27]. This discovery is intriguing because typically, degenerate tissue exhibits a decrease in notochordal/healthy NP markers. However, in this study, it was demonstrated that CD24^+^ cell number increased with age and degeneration, while also positively influencing the expression of notochordal/healthy NP markers. This phenomenon might be attributed to CD24^+^ cells undergoing increased proliferation in response to degeneration to counteract its effects. Another potential explanation may be that of the total number of cells counted a proportion of the CD24^−^ cells are gaining CD24 positivity. Finally, it is recognized that there is a decline in the total number of disc cells with degeneration [33]. Hence, it is also plausible that the rise in CD24 positivity during degeneration and aging stems from a higher rate of death in CD24^−^ cells specifically, leading to a higher proportion of the remaining cells being CD24^+^. It has been suggested that notochordal cells could offer better protection for maintenance of IVD integrity and ECM [34] as well as protection from degradation and apoptosis [13]. Thus, the sub‐populations of CD24 and GD2 positive cells might persist as an attempt at tissue repair or to protect the NP against the effects of degeneration.

In conclusion, this is the first study to investigating the expression of CD24, TIE2 and GD2 at both tissue and cell level in a cohort of pediatric and adult NP samples with aging and degeneration in cells. This study has shown that CD24^+^/GD2^+^ double positive cells can be isolated from adult NP samples of all ages and degeneration grades and this subpopulation of cells can be used for further characterization. It was also shown that CD24^+^ maintain a ‘healthy’ NP phenotype, suggesting that its function in the IVD context should be investigated further.

## Author Contributions

A.M.I. performed experiments, analyzed and interpreted the data and drafted the manuscript; P.B. and S.P. provided support for experiments and data generation; G.H. provided support for the flow cytometry and FACS experiments; J.A.H. and S.M.R. secured funding, contributed to the concept and design the study, interpretation of the data and drafting of the final manuscript. All authors have read and agreed to the published version of the manuscript.

## Conflicts of Interest

J.A.H. is an Editorial Board member of JOR Spine and co‐author of this article. They were excluded from editorial decision‐making related to the acceptance of this article for publication in the journal. All the remaining authors declare no conflicts of interest.

## Data Availability

All data is presented in the manuscript.
